# Cysteine metabolic engineering and selective disulfide reduction produce superior antibody-drug-conjugates

**DOI:** 10.1038/s41598-022-11344-z

**Published:** 2022-05-04

**Authors:** Renée Procopio-Melino, Frank W. Kotch, Amar S. Prashad, Jose M. Gomes, Wenge Wang, Bo Arve, Andrew Dawdy, Lawrence Chen, Justin Sperry, Christine Hosselet, Tao He, Ronald Kriz, Laura Lin, Kimberly Marquette, Lioudmila Tchistiakova, Will Somers, Jason C. Rouse, Xiaotian Zhong

**Affiliations:** 1 BioProcess R&D, Biotherapeutics Pharmaceutical Sciences, Medicinal Sciences, Pfizer Worldwide R&D, 1 Burtt Road, Andover, MA 01810 USA; 2 BioProcess R&D, Biotherapeutics Pharmaceutical Sciences, Medicinal Sciences, Pfizer Worldwide R&D, 875 Chesterfield Parkway West, Chesterfield, MO 63017 USA; 3 Analytical R&D, Biotherapeutics Pharmaceutical Sciences, Medicinal Sciences, Pfizer Worldwide R&D, 875 Chesterfield Parkway West, Chesterfield, MO 63017 USA; 4 Analytical R&D, Biotherapeutics Pharmaceutical Sciences, Medicinal Sciences, Pfizer Worldwide R&D, 1 Burtt Road, Andover, MA 01810 USA; 5 Vaccine Research, Pfizer Worldwide R&D, 401 North Middletown Road, Pearl River, NY 10965 USA; 6 BioMedicine Design, Medicinal Sciences, Pfizer Worldwide R&D, 610 Main Street, Cambridge, MA 02139 USA; 7 Present Address: Pearl River Laboratories Inc, 401 North Middletown Road, Pearl River, NY 10965 USA; 8 Present Address: JOINN Biologics US Inc., 2600 Hilltop Dr., Richmond, CA 94806 USA

**Keywords:** Biological techniques, Biotechnology, Cancer, Cell biology, Chemical biology, Drug discovery, Oncology

## Abstract

Next-generation site-specific cysteine-based antibody–drug-conjugates (ADCs) broaden therapeutic index by precise drug-antibody attachments. However, manufacturing such ADCs for clinical validation requires complex full reduction and reoxidation processes, impacting product quality. To overcome this technical challenge, we developed a novel antibody manufacturing process through cysteine (Cys) metabolic engineering in Chinese hamster ovary cells implementing a unique cysteine-capping technology. This development enabled a direct conjugation of drugs after chemoselective-reduction with mild reductant tris(3-sulfonatophenyl)phosphine. This innovative platform produces clinical ADC products with superior quality through a simplified manufacturing process. This technology also has the potential to integrate Cys-based site-specific conjugation with other site-specific conjugation methodologies to develop multi-drug ADCs and exploit multi-mechanisms of action for effective cancer treatments.

## Introduction

Antibody–drug-conjugates (ADCs) are an important class of biotherapeutics that consist of cytotoxic drugs (known as payloads) covalently linked to an antibody component through a chemical linker^1–7^. Clinically effective ADCs are capable of antigen-specific delivery of highly potent payloads into tumor cells^1–3,8^, as demonstrated by eleven regulator-approved products (Adcetris®, Besponsa®, Blenrep®, Enhertu®, Kadcyla®, Mylotarg®, Padcev®, Polivy®, Tivdax®, Trodelvy®, Zynlonta®). However, clinical trials for many ADCs remain unsatisfactory. Human cancer as a heterogenous disease is typically caused by a diverse population of cells with various gene expression profiles^9,10^. Tumors with low levels or heterogeneity of tumor-associated antigens represent a huge obstacle for achieving truly effective treatment. As part of the search for avenues to improve clinical success rates of ADCs, significant effort has been invested to optimize conjugation site specificity and minimize molecular homogeneity leading to more defined products.

**Figure 1:**
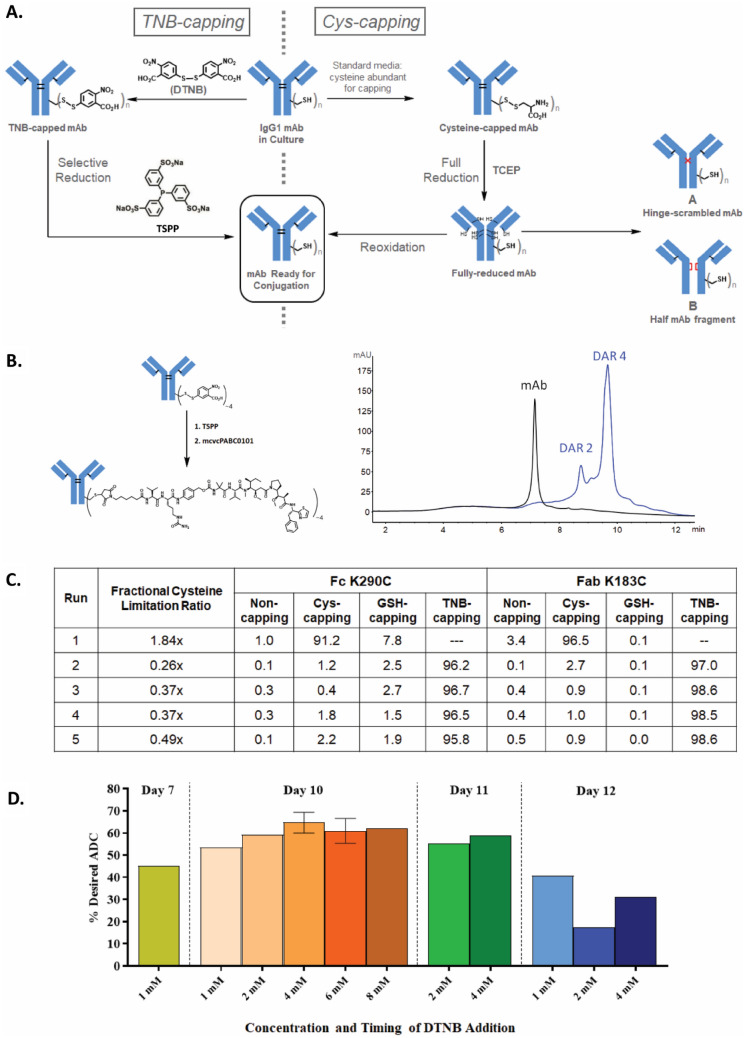
The TNB-capping strategy for chemoselective Cys-based conjugation for manufacturing high-quality clinical-grade site-specific ADCs. (**A**) TNB-capping and Cys-capping pathways to conjugatable antibody. (**B**) TSPP selective-reduction and subsequent direct-conjugation of mcvcPABC0101 linker payload reaction scheme and HIC analysis. TNB-capped Cys-mutant antibody trastuzumab HC-K290C-K334C produced ~ 90% DAR4 ADC. (**C**) Fully TNB-capped Cys-mutant antibody trastuzumab HC-K290C-LC-K183C was generated by stable CHO expression in proprietary basal medium with low fractional cysteine limitation ratios and a 1 mM DTNB bolus addition after the growth phase (Day 7). Conditioned media samples from culture conditions described in the table were purified and the antibody was digested with IdeS and subjected to LC/MS analysis to determine percentages of capping (Non-capped, glutathione (GSH)-capped, Cys-capped and TNB-capped). (**D**) DTNB feed concentrations ranging from 1 to 8 mM were evaluated as bolus additions on Days 7, 10, 11 or 12. The most optimal feed condition, 4 mM DTNB bolus on Day 10, yielded 70% of the desired TNB-capped DAR4 ADC species after TSPP reduction and conjugation.

Homogeneous site-specific high-quality ADCs with the potential for multi-payloads onto a single antibody have been proposed as a novel strategy to improve therapeutic index and to address the key challenge of intratumor heterogeneity and drug-resistance^1,6^. The conjugation for most ADCs target native lysines (Lys) or cysteines (Cys) in the antibodies, resulting in heterogeneous mixtures and limited control over the conjugation site. Cys-based site-specific ADCs allow for precise conjugation to engineered Cys residues and produce a uniform drug-to-antibody ratio (DAR) with potent hydrophobic payloads that otherwise may be incompatible with conventional conjugation^11–13^. Improved therapeutic index and reduced toxicity for Cys-mutant ADCs in preclinical studies as well as the advancement to clinical proof-of-concept^14^ have led to a host of site-specific conjugation strategies in recent years^15–20^. Nevertheless, manufacturing high-quality site-specific ADCs for clinical validation faces major technical challenges. Even for the most extensively investigated Cys-mutant ADCs, a complicated conjugation process is involved, requiring a harsh reduction step to remove the disulfide caps^12,13,21–24^ from the engineered cysteines, and a reoxidation step to reform the disrupted inter-chain disulfides after reduction (Fig. 1A, right pathway). This extensive manipulation not only increases the process complexity, but also introduces disulfide-scrambled non-native modifications (A in Fig. 1A) and half-antibody-fragments (B in Fig. 1A). These drawbacks not only impact drug product quality, but also make the process incompatible with other site-specific methodologies^15,16^ for multi-payload conjugation.

This study developed a potentially revolutionary manufacturing process that produces superior quality next-generation site-specific cysteine-based ADCs by overcoming technical restrictions of the canonic full reduction-reoxidation process. We discovered that Cys metabolic engineering in Chinese hamster ovary (CHO) cells with a unique Cys-capping technology produced antibody suitable direct drug-conjugation after chemoselective-reduction with mild reductant tris(3-sulfonatophenyl)phosphine (TSPP). This innovative platform enables the clinical production of superior quality ADCs with a simplified manufacturing process. This technology enables production strategies compatible with other site-specific conjugation methodologies for potential multi-payload ADCs which should enhance ADC potency, reduce drug-resistance, and improve outcomes for patients.

## Results

### The Cys-capping strategy for chemoselective Cys-based conjugation for manufacturing high-quality clinical-grade site-specific ADCs

To improve manufacturability and ADC quality, we sought a strategy to avoid the full reduction-reoxidation process and reasoned that chemically introducing a labile capping group onto the engineered cysteines is one possible solution. We selected Ellman’s reagent, 5,5’-dithio-bis-(2-nitrobenzoic acid) (DTNB), as the capping reagent, which reacts readily with free thiols, capping them with TNB (Fig. 1A, left pathway). The resulting Cys-TNB-disulfide has a weak redox potential^25^, allowing selective removal of TNB by a mild reductant without disrupting the inter-chain disulfides. Subsequent direct-conjugation to the engineered cysteines would afford the ADC with the native disulfide network intact. The TNB-cap can also serve as a protecting group for the fragile reactive thiol during downstream processing, including purification, concentration, and filtration. This new scheme should not only prevent the introduction of disulfide scrambling but also shorten the conjugation process by eliminating the post-reduction purification and reoxidation steps.

One hurdle to this new strategy is to identify a novel selective reducing agent that can selectively remove the TNB-caps from the antibodies without negatively impacting the protein integrity. The reducing agent used for the current standard process for THIOMAB™^13^ is tris(2-carboxyethyl)phosphine (TCEP) which indiscriminately reduces antibody disulfides due to its high reactivity (Fig. 1A, right pathway)^13^. TCEP also has the potential to desulfurize Cys to alanine at higher concentrations^26^, rendering the site un-conjugatable. Another hurdle is to engineer a novel cell culture process for generating clinical quantities of TNB-capped antibodies. Based on our recent finding that Cys-capping modifications exclusively take place outside mammalian cells during normal cell culture^21^, fully TNB-capped Cys-mutant antibody at research scale in CHO cells was produced through exchange to Cys-free medium. However, Cys was subsequently found essential to CHO proliferation, even with the co-expression of Cys biosynthesis genes^27^, consistent with cysteine’s recent proposed cornerstone role at the origins of life^28^. An innovative approach for the large-scale production of TNB-capped antibody is needed.

### Identifying TSPP as the chemoselective reducing agent for removing TNB-caps

To identify a reducing agent capable of selectively removing TNB-caps, the difference between dialkyl disulfides of interchain disulfides in the antibody and an aryl–alkyl disulfide of the TNB-cap on Cys was exploited. Trialkylphoshines, such as TCEP and tris(3-hydroxypropyl)phosphine, and to a lesser extent, tris(2-cyanoethyl)phosphine, were shown to readily reduce interchain disulfide bonds in an IgG1 antibody even at low temperature (data not shown). This is as expected, since TCEP fully reduces all antibody inter-chain disulfides in the THIOMAB™ process^13^, and tributylphosphine, a highly similar trialkylphosphine, readily reduces a wide range of disulfides^29^. Triphenylphosphine, on the other hand, reduces diaryl disulfides readily, but is much less reactive toward dialkyl disulfides, requiring high temperature and long reaction times to achieve even partial reduction^30^. Most relevant to the selective removal of TNB-caps is a study showing that triphenylphosphine readily reduces small molecule aryl–alkyl disulfides (structural analogs of TNB-cysteine)^31^, so similar triarylphosphines appeared to be ideal reducing agents. Tris(3-sulfonatophenyl)phosphine (TSPP in Fig. 1A), a derivative of triphenylphosphine, has excellent aqueous solubility and is commercially available as a solid trisodium salt, making it suitable for use in biologic manufacturing processes. TSPP was chosen as the selective reducing agent for this process.

To test this hypothesis, an excess of TSPP was used to reduce the TNB-capped trastuzumab heavy chain (HC) L443C- mutant which was previously shown to be fully TNB-capped^21^. The reduced antibody was then conjugated to linker-payload using an excess of mcvcPABC0101 (maleimidocaproyl-valine-citrulline-*para*-aminobenzyloxycarbonyl-0101). As shown in Fig. S1, hydrophobic interaction chromatography (HIC) analysis indicated that TSPP treatment of TNB-capped L443C antibody followed by conjugation yielded a DAR2-ADC with 90% efficiency. Similar results were obtained when TNB-capped HC-K290C-K334C trastuzumab antibody was reduced and directly conjugated to produce DAR4-ADC (Fig. 1B). No over-conjugated species were observed in either experiment, indicating that no interchain disulfides were reduced by this process. These data have demonstrated that a labile protecting group on an antibody, attached through an aryl–alkyl disulfide bond, can indeed be selectively removed using an appropriate reducing agent, leaving the native alkyl-alkyl disulfides in the antibody intact.

### Developing a CHO manufacturing platform for generating TNB-capped antibodies

The next step was to establish a new CHO manufacturing platform for generating TNB-capped materials for clinical development conjugation. The Cys concentration decreased drastically as CHO cells were cultured in bioreactors. It is therefore possible to generate a Cys-free like culture condition through Cys consumption by CHO cells. Unexpectedly, the Cys-capping modification was significantly affected by cell growth and cell density. As shown in Fig. S2A, CHO cells produced substantial uncapped Cys-mutant protein at the cell seed density of 3E6 cells/mL in CD CHO medium (~ 1 mM cystine (Ctn)), whereas fully uncapped Cys-mutant antibody was generated with an increased seed density of 6E6 cells/mL. This implied that most Ctn in the medium was used for cell growth. The increase of cell density can deplete the Ctn in the medium and can consequently affect capping status of the engineered Cys. This observation is consistent with the finding that Cys is an essential amino acid to CHO cells. Cys or Ctn in the culture medium seems preferentially utilized for cell growth presumably through amino acid transporter X_c_^-^ (Fig. S2B)^32^. The Cys-capping reaction outside of the cells appears to be a slow process, and consequentially unpaired surface Cys remains uncapped during high cell density culturing. Another observation is that the viability of the CHO-K1 cells stably expressing the Cys- mutant antibody trastuzumab was not affected by the presence of millimolar range of DTNB (data not shown), indicating DTNB is tolerated by CHO-K1 cells. Since reaction of free thiols in the uncapped antibody with DTNB is a much more rapid chemical reaction than the Cys-capping, it raises the possibility that predominant, if not nearly homogeneous, TNB-capped antibody could be generated in the production bioreactor when the cell culture is exposed to DTNB.

Based on the reasonings above, we hypothesized that we could fine-tune the Cys concentration in CHO cell culture medium during cell growth phase to ensure sufficient Cys is available for cell expansion but limited enough to significantly reduce the Cys-capping reaction on the mutant antibody. When CHO cells reached peak density starting the antibody production phase, various concentrations of DTNB could be added to cell culture for maximal TNB-capping. To accomplish this metabolic engineering strategy, we first determined the impacts of the availability of Cys and/or Ctn to the culture on TNB-capping efficiency. Rational media design and stoichiometric approaches^33^ were used to calculate the required amount of Cys/Ctn needed for a particular peak cell density and amount of product produced (Eq. 1). The fractional cysteine limitation ratio was then calculated to determine the amount of Cys/Ctn needed to be delivered to target a specific limitation ratio, to deliberately limit or deplete the Cys/Ctn in the culture (Eq. 2).

**Equation** (1)**. Required Cysteine Concentration**

1 $${\text{Re}} quired\,\,Cysteine\,\,Concentration = \left[ {\left( {x*m} \right) + \left( {x*m*k} \right) + \left( {p*n} \right)} \right]*f$$

x: cysteine concentration required for E6 cells/mL (0.09 mM).

m: peak cell density ((E6 cells/mL).

k: maintanace factor(10–15%).

p: cysteine concentration required for 1 g/L antibody (019 mM).

n: final antibody concentration (1 g/L).

f: safety factor (1.1–1.3).

**Equation** (2)**. Fractional Cysteine Limitation Ratio**

2 $$Fractional\,\,Cysteine\,\,Limitation\,\,Ratio = \frac{Cysteine\,\,Provided\,\,in\,\,Process}{{{\text{Re}} quired\,\,Cysteine\,\,from\,\,Equation\,1}}$$

As shown in Fig. 1C, different fractional Cys limitation ratio conditions were evaluated in small-scale bioreactors to find a target ratio that limits or depletes Cys/Ctn in order to promote TNB-capping of the antibody while still reaching acceptable peak viable cell densities and harvest titers. In this experiment, the CHO-K1 Cys-mutant HC-K290C-LC-K183C was used with proprietary basal and feed media in 1L-Applikon-bioreactors operated as described in Methods. High-end pH-controlled delivery of glucose (HIPDOG)^34^ was utilized for robust lactate control. For TNB-capping of the antibodies in Runs 2–5, a DTNB bolus was added after the growth phase (Day 7) to target a range of 1 mM DTNB concentration in the bioreactor. Batch duration was approximately 12 days. As shown in Fig. S3, highest peak density achieved was approximately 20E6 cells/mL with > 75% harvest viability for all HIPDOG conditions. Liquid chromatography/mass spectrometry (LC/MS) analysis (Figs. 1C, S4, and S5) on the Cys-mutant HC-K290C-LC-K183C antibody purified from the conditioned medium showed that Runs 2–5 with low fractional Cys limitation ratios all had comparable TNB-capping efficiency to one another, with more than 95.8% at the HC- K290C site and more than 97% at the LC-K183C site. The data demonstrate that TNB efficiently capped the unpaired cysteines of the antibody even with the presence of Cys/Ctn in the cell culture medium.

Next, the concentration and timing of the DTNB feed were evaluated to determine the optimal condition to further maximize TNB-capping efficiency of the antibody, and therefore increase the final percentage of desired DAR4-ADC. In this experiment, an optimized fractional Cys limitation ratio target of approximately 0.8 × was used for TNB-capped conditions. DTNB additions were added as a single bolus on Day 7, 10, 11 or 12 and at a concentration of 1, 2, 4, 6 or 8 mM. All bioreactor conditions were harvested on Day 12, purified, and then treated with TSPP for selective reduction followed by direct conjugation to determine final desired ADC percentage. As shown in Fig. 1D, the optimal DTNB feed condition was an addition of 4 mM DTNB on Day 10 yielding approximately 70% of desired TNB-capped DAR4 species.

### ADC clinical product produced by TNB-TSPP selective-reduction/conjugation demonstrated superior quality

**Figure 2:**
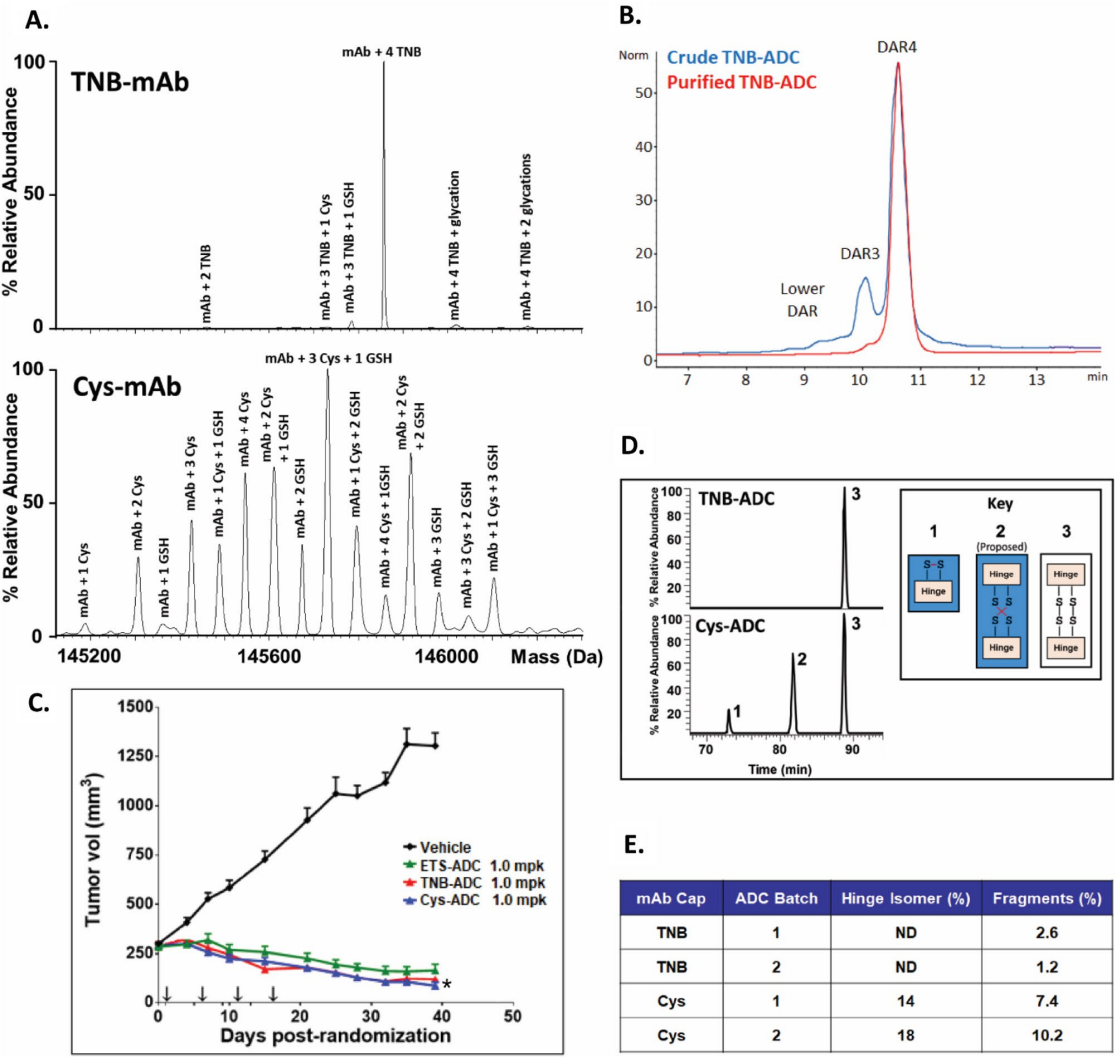
Final antibody generated by TNB-capping and ADC clinical product produced by TSPP selective-reduction/conjugation demonstrate superior quality over those produced by the conventional Cys-capping/conjugation process. (**A**) Intact molecule ESI MS analysis on TNB-capped and Cys-capped HC-K290C-LC-K183C trastuzumab. Peaks are labeled with the capping species (Cys, GSH or TNB) and number of capped locations (i.e., 1, 2, etc.). The TNB-mAb LC/MS analysis shows nearly all antibodies produced had four TNB caps presented, which was the most desired outcome. (**B**) HIC analysis of ADC produced by TSPP reduction of TNB-mAb followed by conjugation to mcvcPABC0101, before and after preparative HIC purification. (**C**) Tumor xenograft data for TNB-ADC, Cys-ADC, and ETS-ADC each dosed at 1 mg/kg Q4Dx4 (arrows on x-axis) after initial staging and randomization in an N87 nude mouse xenograft model. n = 10–15 mice per group. Data are reported as mean ± SEM *P < 0.05 for TNB-ADC as compared to Cys-ADC. (**D**) Extracted ion chromatograms of intrachain and interchain disulfide bonded forms of the hinge region demonstrated the high quality of ADCs produced through TNB-capping strategy versus the Cys-capping strategy. The TNB-ADC showed expected disulfide bond pairing as compared to the Cys-ADC, which required conventional-reduction and re-oxidation, resulting in two disulfide bond mis-paired hinge forms, in addition to the normal interchain disulfide bonding; note that MS/MS sequencing cannot differentiate between the normal (3) and scrambled (2) interchain disulfides because the theoretical fragment ions are the same. (**E**) Product quality comparison of ADCs generated from selective (TSPP)-reduction-conjugation of TNB-capped mAb to ADCs from full (TCEP)-reduction-reoxidation-conjugation of Cys-capped mAb; ADCs produced at 1–4 g scale; Hinge isomer measured by a dedicated non-reduced LysC-IdeS-LC/UV assay and fragments measured by non-reduced capillary gel electrophoresis; data indicate that TNB-ADC was significantly more homogeneous compared to Cys-ADC. Note that it is not expected for the relative quantitation of the LC/MS method (**D**) and the LC/UV assay (**E**) to directly compare due to differences in the detectors and sample preparation.

Since the cell culture process for producing TNB-capped antibody was optimized, the condition of a fractional Cys limitation ratio target of 0.8 × with a 4 mM DTNB bolus addition on Day 10 was executed at large-scale. The large-scale production bioreactor was operated using proprietary basal and feed media in a 130 L stainless steel bioreactor or a 200 L single-use bioreactor. A Cys-capped control, without Cys/Ctn limitation and without a DTNB addition, was also run in parallel. A pH controlled HIPDOG process was utilized for robust lactate control. Cell culture performance was as expected, achieving peak viable cell density and harvest titer within the range demonstrated at small-scale. The resulting antibodies (TNB-capped and Cys-capped control) were purified and analyzed by electrospray ionization mass spectrometry (ESI MS). As shown in Fig. 2A, TNB-capped production process predominantly yields the desired 4TNB-capped-antibody, whereas the Cys-capped production process results in significantly more capping heterogeneity.

The antibodies from the optimized culture process were purified and subjected to the reduction-conjugation process. The yellow color resulting from release of TNB from the antibody can be readily observed almost immediately after TSPP was introduced (Fig. S6). HIC analysis (Fig. 2B, blue trace) indicates that the desired DAR4-ADC was ~ 75% of the crude conjugate mixture. The conjugate can be purified to nearly uniform DAR4-ADC by HIC chromatography (Fig. 2B, red trace). To ensure the TNB-process from mAb through ADC did not impact the efficacy of the final conjugate (TNB-ADC), it was compared directly to the ADC produced from Cys-capped antibody (Cys-ADC) in an in vivo mouse tumor model. Tumor growth curves (Fig. 2C) indicated that TNB-ADC and Cys-ADC were equally efficacious and equivalent to the exploratory-toxicology-material (ETS-ADC), which was also produced from Cys-capped antibody at an earlier stage of development. All three ADCs inhibited tumor growth relative to the untreated control group (Vehicle).

Lastly, the quality of the TNB-ADC and Cys-ADC were compared. Non-reduced LysC digests of the purified ADCs were characterized by LC/MS to examine disulfide bond integrity (Fig. 2D). The Cys-ADC contained 3 different populations of disulfide-bonding in the hinge region: 1. an intrachain disulfide bond which would preclude formation of the normal interchain disulfide bond, 2. a scrambled interchain bond isomer identified by its unique chromatographic retention, and 3. the normal interchain disulfide bonds. The TNB-ADC contained only the normal interchain disulfide bonds. A dedicated non-reduced LysC-IdeS LC/UV assay was developed to specifically monitor hinge region interchain disulfide scrambling. Cys-ADC contains 14–18% hinge-isomer and 7–10% fragmentation whereas TNB-ADC had no detectable hinge scrambling and low-level fragments (Fig. 2E). These data together demonstrate that the TNB-TSPP ADC production strategy, in addition to simplifying the conjugation process, significantly improves product quality for Cys-mutant site-specific ADCs.

## Discussion

In this study, we have described a unique approach for chemoselective conjugation to engineered Cys-mutant site-specific ADCs. This new technology produces ADCs with superior product quality, including homogeneous DAR, native hinge disulfides, and reduced fragments, and has the potential for improving therapeutic efficacy. This platform can be readily adapted to produce site-specific ADCs containing other known Cys-conjugation sites^12,13,22,24^ with minimal modification. Together with oncology indication, this strategy could also be applied to generate site-specific protein-conjugates against other diseases and targets^1,35^.

Besides its immediate application in manufacturing clinical ADCs, the TNB-TSPP strategy demonstrated in this study can be utilized for installation of different payloads onto therapeutic and imaging proteins at defined locations. As shown in Fig. 2A, the Cys metabolic engineering process enabled the generation of nearly homogeneous antibody TNB-capping in the production bioreactor without medium exchange, due to the essentialness of Cys in CHO cell culturing and the inefficiency of Cys-capping. This feasibility in the CHO process manipulation can be applied to produce protein-caps bearing additional chemical handles for conjugation such as biorthogonal click chemistry.

Furthermore, clinical challenges and disease complexities of human cancers demand novel drug payloads, and a single ADC molecule with two or more different payloads may circumvent therapeutic issues of insufficient potency and drug-resistance^1,6^. Currently, Cys-based site-specific conjugation is incompatible with other site-specific conjugation methodologies^15,16^ owing to its limitation of the full reduction-reoxidation process. The TNB-TSPP process developed in this study can overcome these drawbacks by protecting reactive thiols of the engineered Cys in antibodies with TNB during other conjugation reactions, and then selectively removing TNB when ready for subsequent conjugation. Such multi-payload ADCs could open new avenues for future cancer therapies.

## Methods

### Cell line development and protein purification

CHO-K1 cells expressing antibody HC and LC through single-site integration^36^ were grown in CD CHO medium (Thermo Fisher, Waltham, MA) or in-house proprietary medium. Antibody protein purification was performed through rmpProtein A resin (Cytiva, Westborough, MA) and size-exclusion chromatography onto Superdex 200 column in PBS (137 mM NaCl, 2.7 mM KCl, 8.1 mM Na_2_HPO_4_, 2.7 mM KH_2_PO_4_, pH 7.2)^21^. Peak fractions were pooled dialyzed and concentrated into 20 mM Histidine, 8.5% Sucrose, pH 5.8 at 10 mg/mL.

### ESI mass spectrometry

Liquid chromatography mass spectrometry (LC/MS) was performed for intact ADC analysis using a Thermo Exactive Plus EMR mass spectrometer (Thermo Scientific, San Jose, CA) coupled to an Agilent 1260 HPLC (Santa Clara, CA). For de-N-glycosylation, ADCs were treated with PNGase F (Prozyme) at 37 °C for 6 h. The samples were separated over a Thermo MAbPac™ SEC-1, 5 µm, 4 × 300 mm column maintained at 80 °C with a flow rate of 0.2 mL/min. Mobile phases A and B consisted of was 0.05% Trifluoroacetic acid (TFA) in water and 0.05% TFA in acetonitrile, respectively. Proteins were eluted from the column using an isocratic separation: 30% B for 25 min. The mass spectrometer was run in positive mode scanning from 300 to 10,000 m/z and data was acquired with Thermo XCalibur software. The mass spectra signal corresponding to the antibody were summed using Thermo Qual Browser and deconvoluted using Waters MassLynx 4.1 (MaxEnt1) software.

### LC/MS of non-reduced lys-C peptides

A low pH (pH 6.0) non-reduced lysyl endoproteinase Lys-C mapping technique was applied to mAb and ADC samples. The digested samples were acidified with TFA then analyzed by LC/MS on a Thermo LTQ Orbitrap XL coupled to an The Agilent 1260 HPLC. Peptides were separated over an XBridge, BEH130 C18 column (3.5 μm particle sizes, 2.1 mm × 150 mm, Waters PN: 186,003,023) maintained at 60 °C with a flow rate of 0.2 mL/min. Mobile phases A and B consisted of 0.05% TFA in water and 0.05% TFA in acetonitrile, respectively. Peptides were eluted from the column using a gradient designed to recover the expected non-reduced peptides and hydrophobic: 3.0–25.0% B in 22 min, 25.0–34.0% B in 9 min, and 34.0–71.0% B in 37 min. The mass spectrometer utilized internal lock mass ion of hexakis(1H,1H,3H-perfluoropropoxy)phosphazene at m/z 922.009798 for [M + H +]1 + via dynamic calibration. Theoretical molecular masses for peptides were calculated using Bruker Sequence Editor. The observed multiply-charged peptides were converted to neutral molecular masses using Xtract, a software component of Thermo Qualbrowser. Neutral, monoisotopic masses were reported for all peptides observed in the LC/MS data and UV absorbance was monitored at 214 nm.

### Cell culture process generating TNB-capped and Cys-capped Cys-mutant antibodies

A CHO cell line derived from the host CHO-K1, were used for all cell culture experiments. Cells were pre-adapted to serum-free suspension growth prior to transfection. Cells were stably transfected with proprietary DNA vectors to express a cysteine mutant monoclonal antibody. Cultures were scaled-up and maintained in humidified incubator conditions of 5% CO_2_ at 37 °C. Production of the TNB-capped antibody was executed in small-scale 1 L bioreactors (Applikon, Inc., Schiedam, Netherlands), or at large-scale in a 130 L stainless steel bioreactor or a 200 L single-use bioreactor. The production cell culture process utilized HIPDOG^34^. Dissolved oxygen was controlled at > 20% of air saturation by sparging of pure oxygen, temperature was controlled at 37 °C, and pH was maintained near 7.0 using a basic titrant and either CO_2_ or glucose containing nutrient feed. All media and feeds used are proprietary solutions developed by Pfizer; production media and feeds varied in cysteine and/or cystine concentrations for experimental purposes. Ellman’s reagent DTNB was added as a one-time bolus to production bioreactors of experimental conditions at various concentrations (1–8 mM) and timepoints (Days 7, 10, 11, or 12). Production of the Cys-capped antibody was executed in small-scale 1 L bioreactors (Applikon, Inc., Schiedam, Netherlands), or at large-scale in a 130 L stainless steel bioreactor or a 200 L single-use bioreactor. The production cell culture process utilized HIPDOG control. Dissolved oxygen was controlled at > 20% of air saturation by sparging of pure oxygen, temperature was controlled at 37 °C, and pH was maintained near 7.0 using a basic titrant and either CO_2_ or glucose containing nutrient feed. All media and feeds used are proprietary solutions developed by Pfizer; production media and feeds were not limited in cysteine/cystine concentrations and no DTNB feed was added to the production bioreactor. All conditions were harvested after 12 days. Large-scale production runs were clarified by centrifugation prior to downstream processing to generate drug substance intermediates.

### Drug substance intermediate purification process

The MabSelect Protein A affinity (Cytiva Uppsala, Sweden) chromatography column was equilibrated with 50 mM Tris, 150 mM NaCl, pH 7.5 prior to loading. Clarified condition media was then applied followed by a two column volumes (CV) wash of the equilibration buffer. This was followed by 5 CV’s of 50 mM Tris, 0.5 M Calcium Chloride, pH 7.5 and 5 CV’s of 10 mM Tris, 10 mM NaCl, pH 7.5. The protein is eluted with 150 mM Glycine, 40 mM NaCl, pH 3.5. The elution pool consisted of material collected from start in UV rise, to a total of 3 CV’s, collected as the process pool. The remaining bound protein was removed using an additional 5 CV’s of 50 mM Glycine, 250 mM NaCl, pH 2.7 followed by sanitization with 50 mM NaOH, 0.5 M sodium sulfate. Fractogel® EMD TMAE HiCap (M) AEX (EMD Merck Darmstadt, Germany) column was equilibrated with 50 mM Hepes, 65 mM NaCl, pH 7.0. Protein A peak pool was applied to the column followed by a 3 CV wash of the equilibration buffer. The load eluate and wash volumes were collected together as the process pool, and any remaining bound protein was removed using a 50 mM Tris, 2 M NaCl strip buffer. The columns were sanitized with 2 M NaCl, 0.5 M NaOH. The product pool from the TMAE HiCap chromatography step is filtered through an equilibrated single-use virus retaining filter cartridge (Millipore, Viresolve Pro). The product is collected in the permeate stream. The VRF pool is subjected to UF/DF for concentration and buffer exchange (Millipore PLCTK, 30KD membrane). The filtrate is concentrated to a preset volume. The concentrate is diafiltered for 7 diavolumes into 22 mM histidine, pH 5.7 buffer and then further concentrated to achieve target concentration of 50 g/L.

### TSPP reduction and conjugation process at small scale

The TNB-capped conjugation protocol consists of two steps leading to the crude conjugate: selective reduction, and conjugation. In the first step a selective reduction of TNB-capped cysteines (but not interchain disulfides) is accomplished, typically using an excess (~ 10 equivalents) of TSPP at 25 °C for 2 h. In a specific instance: To 1 mg (6.9 nmol; 7.6 mg/mL in PBS; 131.58 µL) of TNB-capped HC-K290C/K334C antibody in a 0.5 mL eppendorf tube was added 39.2 µg of TSPP (10 equivalents; 69.05 nmol; 50 mM in water; 1.38 µL). The reaction mixture was incubated at 25 °C for 2 h to produce reduced antibody. In the second step the unprotected mutant cysteines are conjugated, typically with an excess (~ 10 equivalents) of linker-payload at 25 °C for 1 h to produce the crude conjugate. In a specific instance: To the reduced antibody mixture above was added 92.6 µg of mcvcPABC0101 linker-payload (10 equivalents, 69.05 nmol; 10 mM in dimethylsulfoxide; 6.9 µL). The reaction mixture was incubated at 25 °C for 1 h to afford crude conjugate.

### TNB-ADC batch 1

To 1.0 g (6.9 µmol; 25 mg/mL in 60 mM histidine, pH 7; 38.9 mL) of K183C/K290C antibody was added 27.5 mg of TSPP (7 equivalents; 48.3 µmol; 10 mM in water; 4.83 mL). The reaction mixture was incubated at ambient temperature for 2 h. To this reaction mixture was added 111 mg of mcvcPABC0101 linker-payload (12 equivalents, 82.7 µmol; 25 mM in dimethylsulfoxide; 3.31 mL). The reaction mixture was incubated at ambient temperature for 1 h to afford crude conjugate.

### TNB-ADC batch 2

To 4.0 g (27.6 µmol; 25 mg/mL in 60 mM histidine, pH 7; 156 mL) of K183C/K290C antibody was added 93.8 mg of TSPP (6 equivalents; 165 µmol; 10 mM in water; 16.5 mL). The reaction mixture was incubated at 37 °C for 3 h, and then buffer exchanged by diafiltration (TangenX ProStream 50kD membrane, 110–210 g/m^2^, 10 diavolumes of 60 mM histidine, pH 7). Following diafiltration, 222 mg of mcvcPABC0101 linker-payload (6 equivalents, 165 µmol; 25 mM in dimethylsulfoxide; 6.62 mL) was added. The reaction mixture was incubated at 25 °C for 1 h to afford crude conjugate. Note that for this batch, a diafiltraiton step was added only to accommodate a reduced amount of linker-payload compared to Batch 1.

### Cys-ADC batch 2

To 4.0 g (27.6 µmol; 27 mg/mL in 60 mM histidine, pH 7; 144 mL) of cysteine-capped K183C/K290C antibody was added 1.65 mL of 0.5 M TCEP (30 equivalents; 0.827 mmol; 0.5 M solution in water). The reaction mixture was incubated at 37 °C for 5 h, and then buffer exchanged by diafiltration (TangenX ProStream 50kD membrane, 110–210 g/m^2^, 10 diavolumes of 60 mM histidine, pH 7). Following diafiltration, the mixture was cooled to 4 °C, 0.144 g dehydroascorbic acid (30 equivalents; 0.827 mmol; 50 mM in 1:1 DMSO/water; 16.5 mL) was added, and the mixture was incubated at 4 °C for approximately 16 h. The mixture was heated to 25 °C, 0.185 g of mcvcPABC0101 linker-payload (5 quivalents, 0.138 mmol; 25 mM in dimethylsulfoxide; 5.51 mL) was added, and the mixture was incubated at 255 °C for 1 h to afford crude conjugate.

### Cys-ADC batch 1

Produced by the same protocol as Cys-ADC Batch 2, except starting with 1.0 g of Cys-capped HC-K290C-LC-K183C antibody.

### Antibody subunit liquid chromatography mass spectrometry analysis

Antibody samples were digested with PNGase F (New England BioLabs, Ipswich, MA) to remove Fc-glycans. Subsequent IdeS (Genovis Inc, Cambridge, MA) digestion separated the Fab2 from the scFc. The samples were acidified by diluting 1:1 with 0.05% TFA (Sigma-Aldrich, St. Louis, MO) and analyzed by LC/MS on a Waters Xevo Q-TOF G2 mass spectrometer (Waters, Milford, MA) coupled to an Agilent (Santa Clara, CA) 1200 capillary HPLC. The deglycosylated subunits were separated over a Waters BEH300 C4, 1.7 µm, (1.0 × 50 mm) column maintained at 80 °C with a flow rate of 65 µl/min. Mobile phases A and B consisted of water with 0.05% TFA, and acetonitrile with 0.05% TFA, respectively. Proteoforms were eluted from the column using a gradient: 2 to 20% B in 0.5 min, 20–40% B in 6 min, and 40–100% B in 4 min. The mass spectrometer was run in positive MS only mode scanning from 800 to 3500 m/z and data was acquired and spectra summed and deconvoluted (MaxEnt1) using Waters MassLynx 4.1 software.

### Non-reduced capillary gel electrophoresis for final ADCs

Capillary Gel Electrophoresis was performed on a Beckman PA 800 Plus. ADC sample was diluted to 1 mg/mL into a 60 mM Tris buffer at pH 6 in the presence of 0.5% SDS and 12.5 mM iodoacetamide. The sample was then mixed by vortex and heated for 10 min at 68 °C in a water bath. After heating, the ADC sample was transferred to a microvial and loaded into the Beckman PA 800 Plus. Samples are electrokinetically injected for approximately 20 s into a 50 µm bare fused silica capillary of 10 cm effective length that is pretreated with the Beckman SDS gel separation buffer. 15 kV is applied across the capillary for 20 min to achieve separation of monomer and fragment species monitored by UV detection at 220 nm.

### Assay to analyze disulfide scrambling at the hinge region of the antibody

1 mg/mL ADC samples were treated with IdeS (1 unit/mg ADC, cleaves below hinge) at 37 °C for 30 min followed by treatment with LysC (1 mg/150 mg ADC, cleaves above hinge) at 37 °C for 5 min. Reactions were quenched with trifluoroacetic acid. The samples were analyzed by LC/UV/MS on a Thermo Exactive Plus EMR mass spectrometer coupled to an Agilent (Santa Clara, CA) 1200 capillary HPLC. The hinge proteoforms were separated over a Waters XBridge BEH C18 column maintained at 60 °C with a flow rate of 0.2 mL/min. Mobile phases A and B consisted of water with 0.1% TFA, and acetonitrile with 0.085% TFA, respectively. Proteoforms were eluted from the column using a gradient: 20–30% B in 30 min. The relative abundance of the hinge proteoforms was quantified using UV absorbance (214 nm), and identities were confirmed by mass spectrometry.

### Analytical HIC on ADCs

The following experimental procedure is for the HIC analysis to determine ADC drug loading and distribution shown in Fig. 1B. Other HIC analyses used this method or slight variations. Test samples were diluted to 1–2 mg/mL with phosphate-buffered saline and injected onto a TOSOH TSKgel® Butyl-NPR column (4.6 mm × 3.5 cm, 2.5 um). Mobile phase A was 1.5 M ammonium sulfate, 50 mM potassium phosphate, pH 7, and mobile phase B was 20% (v/v) isopropanol, 50 mM potassium phosphate, pH 7. Injection amounts were typically in the range of 25–75 mg protein. Species were eluted at a flow rate of 0.8 mL/min with a gradient of 0–100% mobile phase B over 12 min and monitored by UV detection at 280 nm. The average DAR and distribution profile were determined by the peak area percentage of each species.

### In vivo mouse tumor xenograft study

All animal studies were approved by the Pfizer Institutional Animal Care and Use Committee (IACUC) according to established guidelines. The animal study used in the reporting of this manuscript follows the recommendations in the ARRIVE guidelines^37^.

Female athymic nude mice (Charles River Laboratories stock no.: 088) were implanted subcutaneously with 7.5 × 10^6^ N87 cells in 50% Matrigel (BD Biosciences). Mice were randomized into study groups of 10–15 mice each when tumors reached approximately 300 mm^3^. Either test agents ETS-ADC, TNB-ADC, Cys-ADC each at 1 mg/kg, or PBS (Gibco, catalog no., 14,190–144, as vehicle) were administered intravenously starting on day 0 for a total of four doses, 4 days apart (Q4Dx4). Tumors were measured at least weekly with a Vernier caliper (Mitutoyo) and the tumor mass was calculated as volume (mm^3^) = (width × width × length)/2. In the tumor growth curves, tumor volume is plotted as mean tumor volume ± SEM. Animal body weights were measured and recorded at least once a week. When individual tumor volume approached 15% of the initial body weight, or other IACUC compliant endpoints were reached, the mouse was humanely euthanized with CO2 following the IACUC approved protocols.

## Supplementary Information

Supplementary Information.
