# Supplementary material for: Cysteine metabolic engineering and selective disulfide reduction produce superior antibody-drug-conjugates
Source: Sci Rep. 2022 May 4;12:7262. doi: 10.1038/s41598-022-11344-z (PMC9068625; doi:10.1038/s41598-022-11344-z)
Supplement: Supplementary file 1 — Supplementary Information. [file 41598_2022_11344_MOESM1_ESM.pdf]

Supplementary Materials for

Cysteine Metabolic Engineering and Selective Disulfide Reduction Produce Superior Antibody-Drug-Conjugates

Renée Procopio-Melino[1#], Frank W. Kotch[2#¶], Amar S. Prashad[2#], Jose M. Gomes[1#¶], Wenge Wang[1], Bo Arve[2], Andrew Dawdy[3¶], Lawrence Chen[4], Justin Sperry[3], Christine Hosselet[5], Tao He[6], Ronald Kriz[6], Laura Lin[6], Kimberly Marquette[6], Lioudmila Tchistiakova[6], Will Somers[6], Jason C. Rouse [4], and Xiaotian Zhong[6¶]

[1]Bioprocess R&D, Pharmaceutical Sciences, Pfizer Inc. 1 Burtt Road, Andover, MA 01810. [2]Bioprocess R&D, Pharmaceutical Sciences, Pfizer Inc. 875 Chesterfield Parkway West, Chesterfield, MO 63017. [3]Analytical R&D, Pharmaceutical Sciences, Pfizer Inc. 875 Chesterfield Parkway West, Chesterfield, MO 63017. [4]Analytical R&D, Pharmaceutical Sciences, Pfizer Inc. 1 Burtt Road, Andover, MA 01810. [5]Vaccine Research, Worldwide R&D, 401 North Middletown Road, Pearl River, NY10965. [6]BioMedicine Design, Worldwide R&D, Pfizer Inc. 610 Main Street, Cambridge, MA 02139. [#]Equal contributors. [¶]Correspondence: Frank.W.Kotch@pfizer.com, Jose.Gomes@pfizer.com, Andrew.W.Dawdy@pfizer.com, Xiaotian.Zhong@pfizer.com.

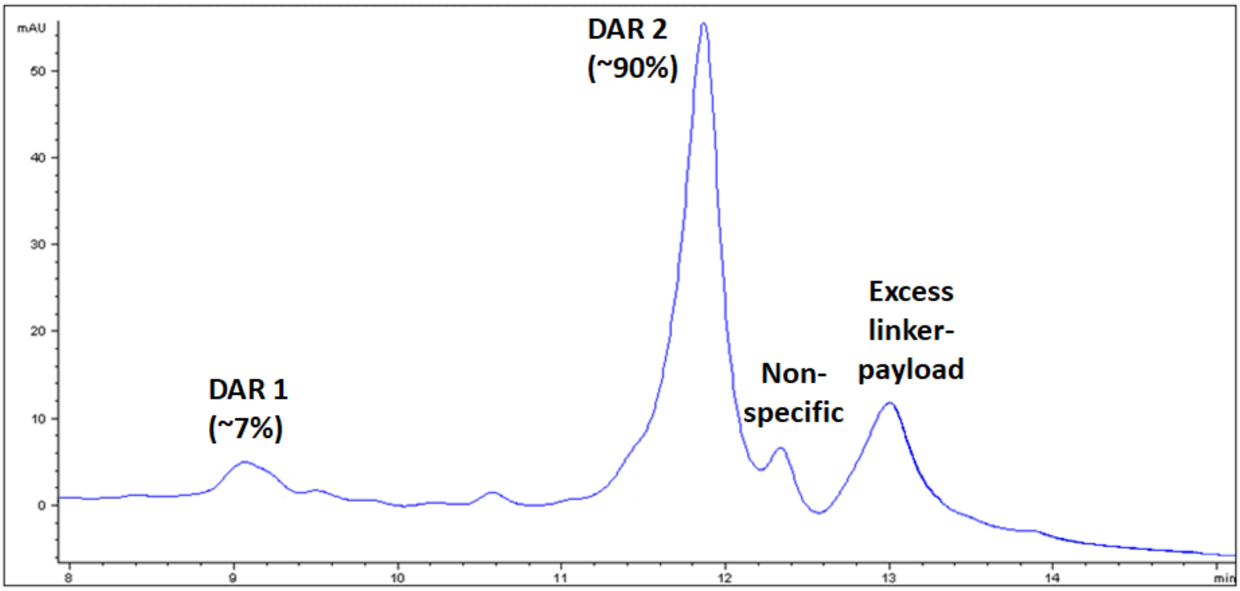

Figure S1. TSPP selective reduction of TNB-capped trastuzumab HC-L443C mutant antibody followed by conjugation to mcvcPABC0101 linker payload produced 90% DAR2 ADC. HIC analysis shows the DAR distribution. The non-specific peak is likely a DAR2 ADC with mcvcPABC0101 conjugated to an alternative cysteine residue. The excess linker payload peak identity was confirmed by comparison to injection of mcvcPABC0101 alone.

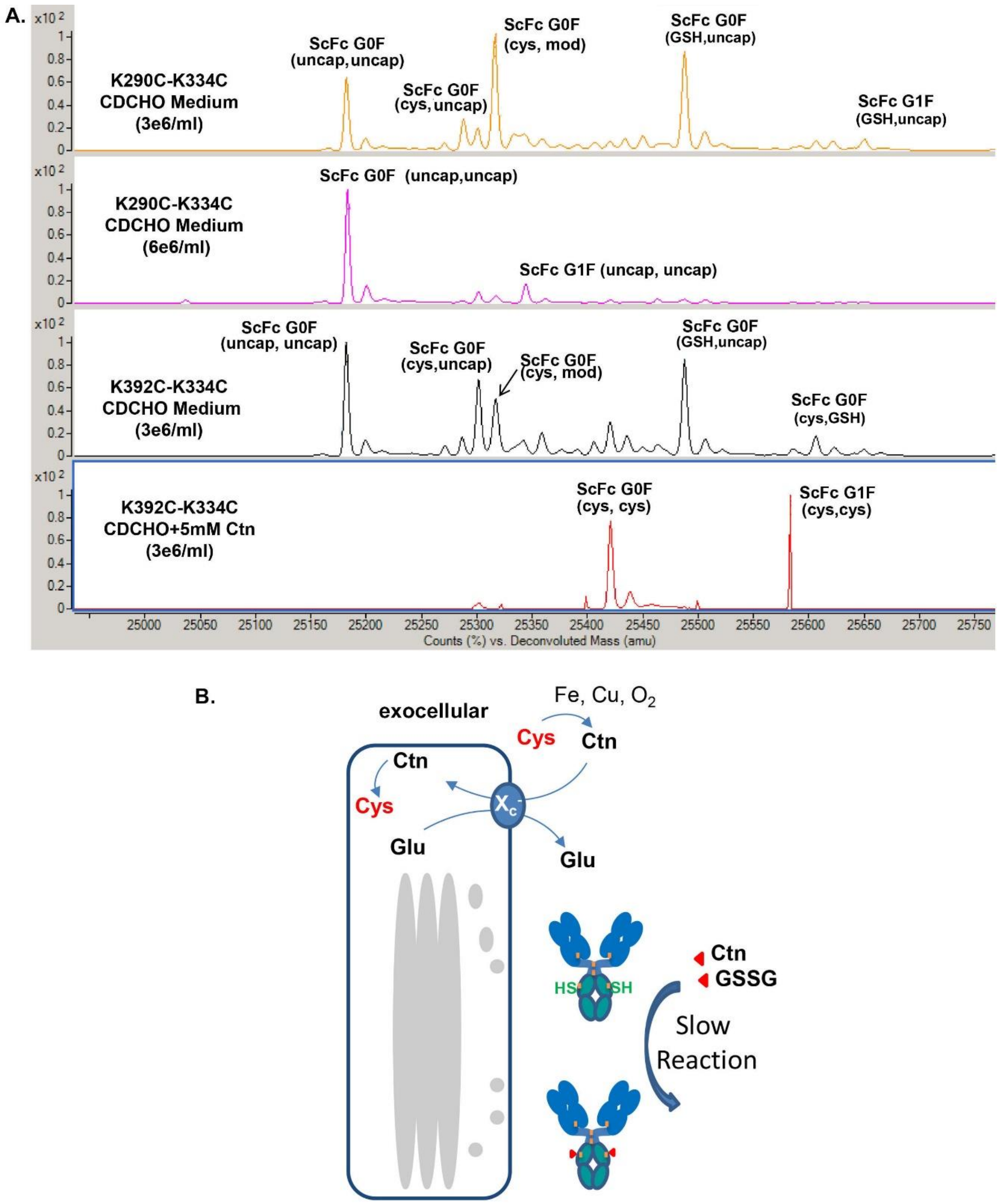

Figure S2. Fully uncapped Cys mutant antibody was generated by high cell density culture of stable CHO expression in regular CD CHO medium. CHO-K1 cells, stably expressing trastuzumab Cys mutant HC-K290C-K334C or HC-K392C-K334C, were seeded in CD CHO medium with the density of 3E6 cells/mL or 6E6 cells/mL and cultured for 72 hours at 37°C. Conditioned media were purified through Protein A and size-exclusion chromatography. A. Purified antibody proteins were subjected to mass spec analysis. B. Cellular model for Cys metabolism. Cys and its oxidized form cystine (Ctn) in the culture medium is preferentially utilized for cell growth because Cys is an essential amino acid to animal cells. Ctn is transported into cytoplasm presumably through amino acid transporter X_c^- exchanged with glutamate (Glu). The Cys-capping reaction by Ctn/Cys/Glutathione (GSH, GSSG) outside of cells is a slow process, and consequentially unpaired surface Cys remains uncapped with free thiols during high cell density culturing.

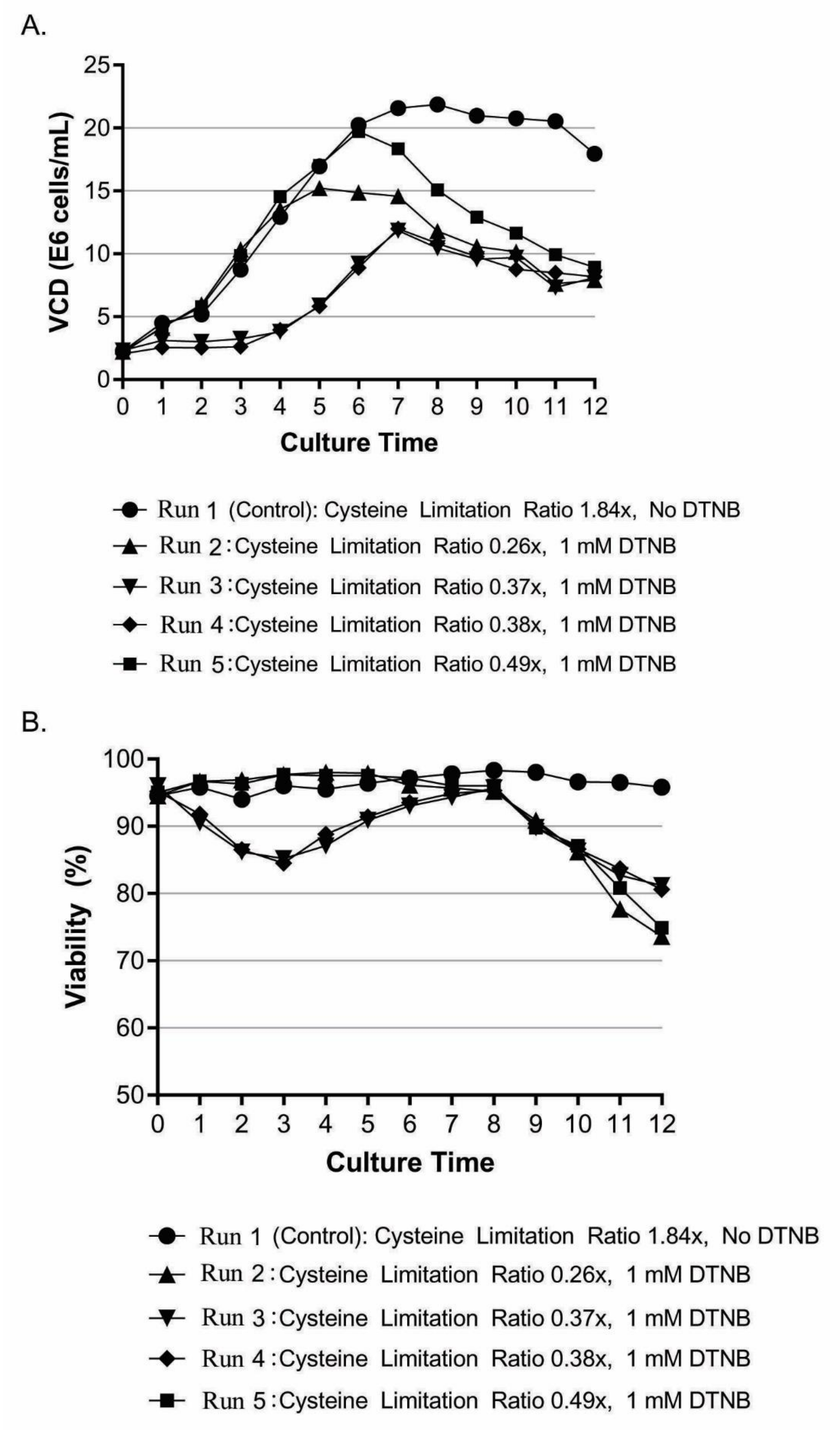

Figure S3. Cell culture performance of stable CHO expression in proprietary basal medium with low fractional cysteine limitation ratios and a 1 mM DTNB bolus addition after the growth phase (Run 2 – Run 5), with the exception of the control (Run 1) that did not receive the DTNB addition. All conditions were seeded at 2E6 cells/mL in a controlled bioreactor, utilizing HIPDOG for robust lactate control. Panel A shows viable cell density (VCD) and Panel B shows viability.

A.

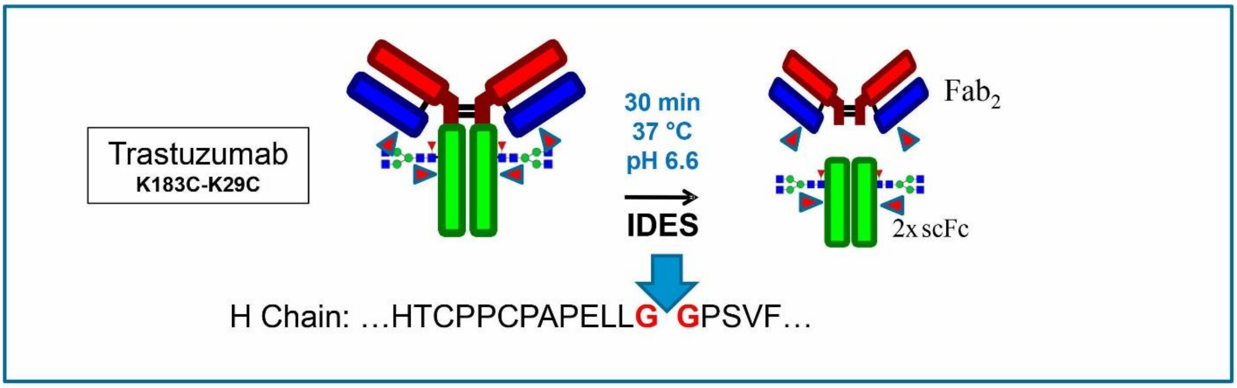

B.

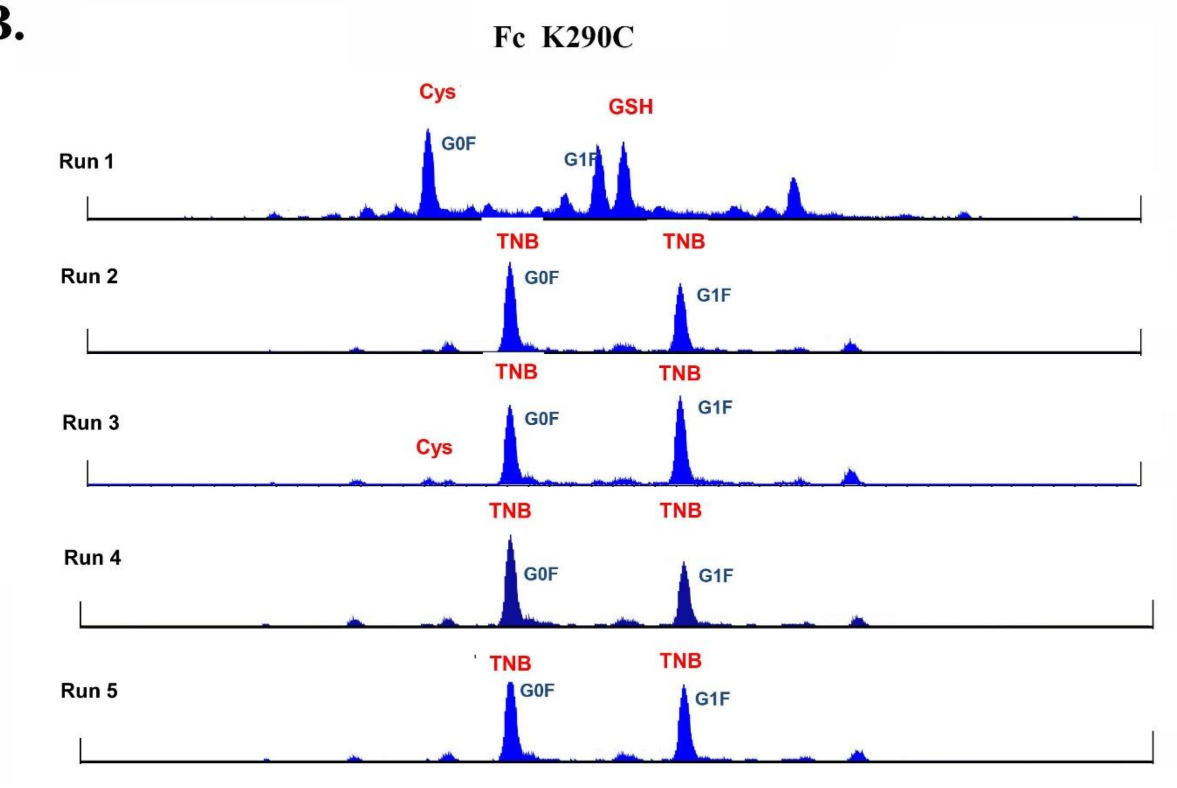

Figure S4. Mass spec analysis on fully TNB-capped Cys mutant antibody at Fc K290C site generated by high cell density of stable CHO expression in CHO medium with DTNB. Conditioned media from culture conditions in Figure S3 were purified through Protein A and size-exclusion chromatography. A. The trastuzumab HC-K290C-LC-K183C mutant antibody was digested with IDES. B. Digested antibody was subjected to LC-MS/MS analysis.

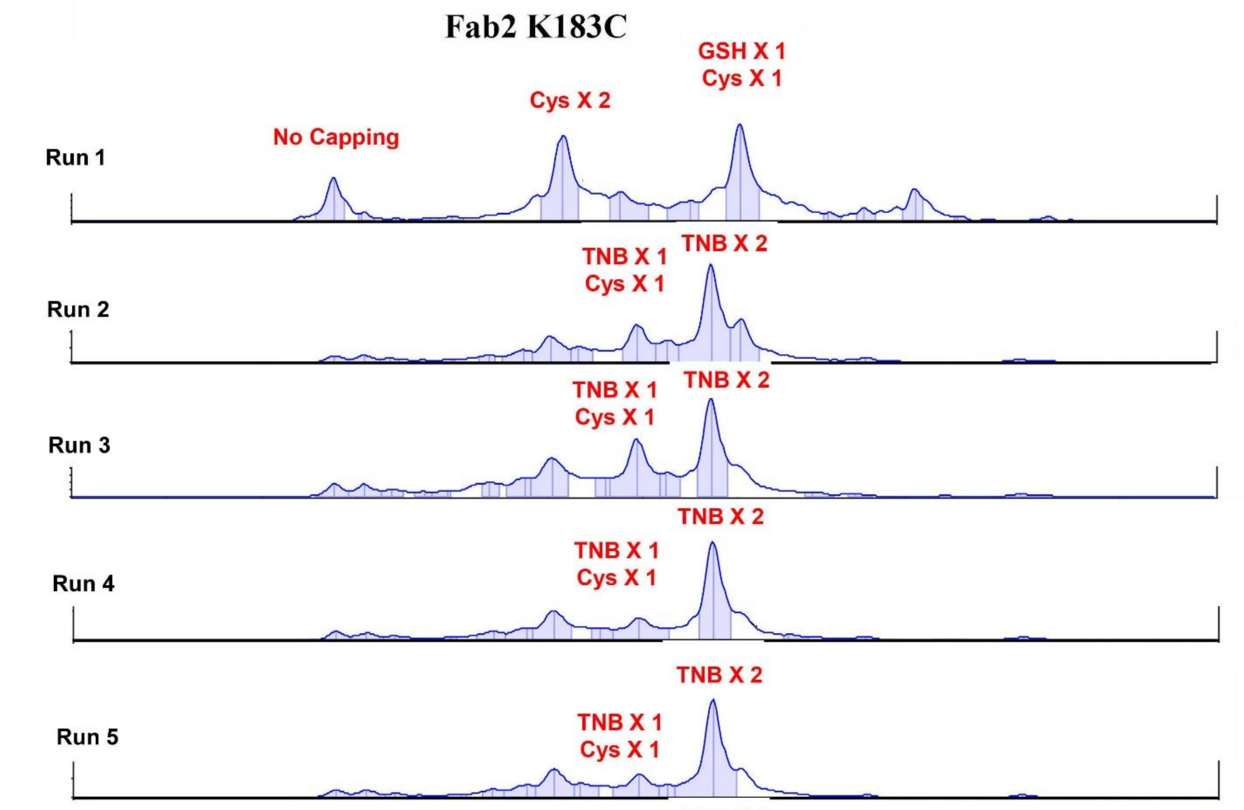

Figure S5. Mass spec analysis on fully TNB-capped Cys mutant antibody at Fab LC-K183C site generated by high cell density of stable CHO expression in CHO medium with DTNB. Conditioned media from culture conditions in Figure S3 were purified through Protein A and size-exclusion chromatography. The trastuzumab HC-K290C-LC-K183C mutant antibody was digested with IDES (Figure S4A) and subjected to LC-MS/MS analysis.

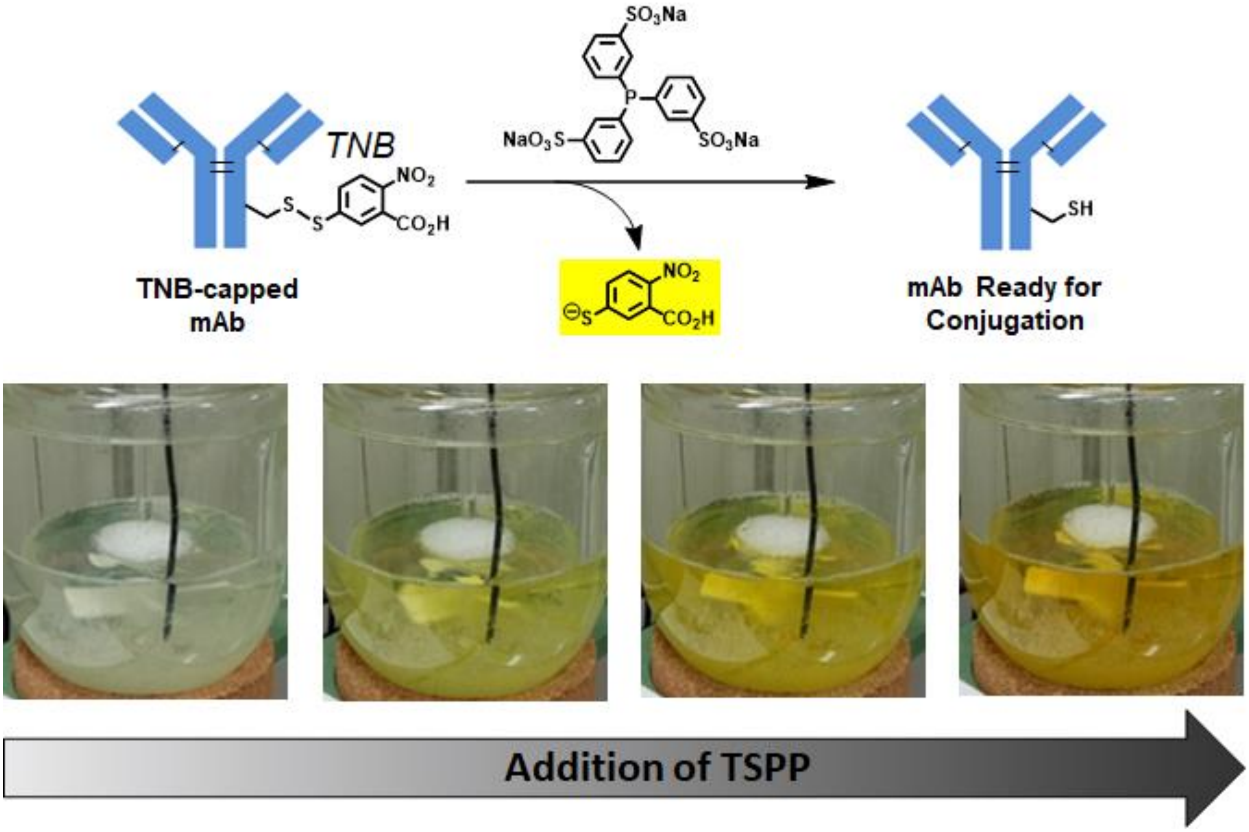

Figure S6. Color change during early addition of TSPP at the start of TNB-mAb reduction.
